# Effects of Solvent and Electrospinning Parameters on the Morphology and Piezoelectric Properties of PVDF Nanofibrous Membrane

**DOI:** 10.3390/nano12060962

**Published:** 2022-03-14

**Authors:** Jia-Yi Yin, Carlo Boaretti, Alessandra Lorenzetti, Alessandro Martucci, Martina Roso, Michele Modesti

**Affiliations:** Department of Industrial Engineering, University of Padova, Via Marzolo, 9, 35131 Padova, Italy; jiayi.yin@phd.unipd.it (J.-Y.Y.); carlo.boaretti@unipd.it (C.B.); alessandra.lorenzetti@unipd.it (A.L.); alex.martucci@unipd.it (A.M.); modesti.michele@unipd.it (M.M.)

**Keywords:** PVDF, nanofibrous membrane, electrospinning, piezoelectric properties

## Abstract

PVDF electrospun membranes were prepared by employing different mixtures of solvents and diverse electrospinning parameters. A comprehensive investigation was carried out, including morphology, nanofiber diameter, crystallinity, β-phase fraction, and piezoelectric response under external mechanical strain. It was demonstrated that by using low-toxicity DMSO as the solvent, PVDF membranes with good morphology (bead-free, smooth surface, and uniform nanofiber) can be obtained. All the fabricated membranes showed crystallinity and β-phase fraction above 48% and 80%, respectively; therefore, electrospinning is a good method for preparing PVDF membranes with the piezoelectric properties. Moreover, we considered a potential effect of the solvent properties and the electrospinning parameters on the final piezoelectric properties. When PVDF membranes with different β-phase fractions and crystallinity values are applied to make the piezoelectric transducers, various piezoelectric voltage outputs can be obtained. This paper provides an effective and efficient strategy for regulating the piezoelectric properties of PVDF electrospun membranes by controlling both solvent dipole moment and process parameters. To the best of our knowledge, this is the first time that the influence of a solvent’s dipole moment on the piezoelectric properties of electrospun materials has been reported.

## 1. Introduction

Piezoelectric materials, which are extensively used in energy harvesting and sensors, have attracted much attention recently [1,2]. In brief, piezoelectric material can convert external mechanical strain into electric energy and vice-versa. Piezoelectric materials can be divided into three main categories: single crystals (e.g., quartz crystal), ceramics (e.g., lead zirconate titanate, BaTiO_3_), and polymers (e.g., poly(vinylidene fluoride) (PVDF) and its copolymers, polylactic acid and polyimides) [3]. Among them, piezoelectric polymers present specific advantages, such as light weight, deformability, and flexibility; therefore, they have the potential to be employed as stretchable and flexible electronics [3,4,5].

PVDF has become the most widely investigated piezoelectric polymer because of its excellent properties and low price [4,6,7]. PVDF is a polymorphic material and has distinct chain conformations in five crystalline phases: TTT (all-trans) planar zigzag for β phase, TGTG’(trans-gauche-trans-gauche) for α and δ phases, and T_3_GT_3_G’ for γ and ε phases [8]. A strong electrical dipole moment exists in the PVDF monomer because the fluorine atom is more electronegative than hydrogen and carbon atoms, and it leads to piezoelectric properties. From the packing model of PVDF chain conformation, it can be concluded that there is no net dipole in α and ε phases, but there are net dipoles in β, γ, and δ phases [9]. Among these three phases, the β phase has the highest dipolar moment per unit cell and endues PVDF with the greatest piezoelectricity [10,11].

The essence of improving the piezoelectric properties of PVDF is to improve the alignment of chain conformation. The main methods to improve the piezoelectric properties of PVDF are: (a) adding treatments, such as melt quenching [12] and stretching [13]; (b) blending with carbon materials [14,15], inorganic particles [16], piezoelectric ceramics [17], etc.; (c) adopting new process methods, such as electrospinning [18]; and (d) altering the structure [19] or surface morphology [11,20].

Electrospinning is a simple and versatile method for producing fiber membranes with a fiber diameter on the nanometer scale. It consists of three essential parts: a high-voltage supply, a spinneret with the polymer solution, and a grounded collector. A suitable solution is a key point for electrospinning. The polymer could be dissolved in a suitable solvent or melted at a high temperature to obtain a homogeneous and flowable polymer solution. Additionally, proper viscosity, surface tension and conductivity are mandatory for solutions undergoing electrospinning. Under proper conditions, which means suitable processing parameters and ambient parameters, the polymer solution can form a stable Taylor cone at the tip of the spinneret. Then, the Taylor cone erupts to the collector when its electrostatic repulsion is equal to or higher than its surface tension. A nanofibrous membrane can be gathered on the collector, and its thickness depends on the electrospinning time. Processing parameters (voltage, tip-to-collector distance, and feed rate) and ambient parameters (humidity and temperature) influence the morphology and structure of the fiber.

As one of the most highly fluorinated polymers, PVDF is resistant to many standard organic solvents. In order to consume less time and effort finding soluble solvents for polymers, including PVDF, before the actual dissolution experiment, the Hansen solubility parameters [21] were introduced, which define the solubility of a polymer–solvent system. The radius of interaction (*R_o_*) of a polymer defines a solubility sphere and is empirically calculated. $\delta_{d}$, $\delta_{p}$, and $\delta_{h}$ are the dispersion, polar, and hydrogen-bonding solubility parameters, respectively. Solvents with Hansen parameters within *R_o_* can dissolve the polymer. The distance (*R_a_*) between the solvent coordinate and the center of the polymer solubility sphere is calculated according to Equation (1):

(1) $$R_{a}=\sqrt{4{\left(\delta_{d}^{p}-\delta_{d}^{s}\right)}^{2}+{\left(\delta_{p}^{p}-\delta_{p}^{s}\right)}^{2}+{\left(\delta_{h}^{p}-\delta_{h}^{s}\right)}^{2}}$$

where $\delta_{d}^{p}$, $\delta_{p}^{p}$, and $\delta_{h}^{p}$ are Hansen parameters for the polymer, and $\delta_{d}^{s}$, $\delta_{p}^{s}$, and $\delta_{h}^{s}$ are Hansen parameters for the solvent [21,22]. The ratio *R_a_/R_o_* is called the relative energy difference (RED). The polymer can be dissolved in a solvent when *R_a_*/*R_o_* < 1, whereas a solvent cannot dissolve the polymer when *R_a_*/*R_o_* > 1 [22]. Bottino et al. [23] a solubility experiment of 46 kinds of solvents on PVDF and obtained the Hansen space of PVDF, which provided a direct criterion for judging the solubility of PVDF in each solvent.

Solubility supplies the possibility of preparing PVDF solution, which is a basic step in electrospinning a nanofibrous membrane. In addition, some properties of the solvents have effects on the final membrane morphology: evaporation rate influences not only the morphology but also the piezoelectric properties of cast film and electrospun membrane, as reported by Kim et. al. [24]. Moreover, the high dipole moment of the solvent is the main reason for the better piezoelectric properties of cast films [25,26,27]. Solution conductivity is also a key factor in controlling the diameter of nanofibers [28].

The boiling point of the solvent corresponds to the temperature when the vapor pressure of the liquid is equal to the environmental pressure and is used to reflect the evaporation rate. A higher boiling point means a slower evaporation rate and vice-versa. Kim et al. [24] used three different solvents (DMF, DMF/ACE (6/4), and MEK) to prepare P(VDF-TrFE) electrospun nanofibrous membranes and studied the effects of these solvents on crystallization, fiber formation, and harvesting performance. They found that two key solvent properties (surface tension and evaporation rate) can affect the fiber diameter, degree of crystallization, and β-phase content.

Marcel Benz et al. [25] prepared PVDF cast films with different solvents and found that the PVDF films with a γ phase (polar phase) can be produced when the solvent has a high dipole moment. G. Knotts et al. [26] studied the influence of solvents (DMSO, DMF, and MEK) on the ferroelectric properties of PVDF-TrFE spin-cast film and found the film prepared with DMSO (highest dipole moment among three solvents) had excellent ferroelectric output. Kim et al. [27] conducted a detailed and comprehensive work on the piezoelectric properties of P(VDF-TrFE) films using solvents with different dipole moments. They found solvents with a high dipole moment can lead to a P(VDF-TrFE) film with high piezoelectric and pyroelectric coefficients, as well as triboelectric properties.

The Hansen solubility parameters and the physical properties of the common soluble solvents for PVDF are listed in Table 1. It can be seen that DMF and DMSO with high dipolar moments are good solvents for PVDF, whereas THF and ACE with low dipolar moments are swelling solvents for PVDF [23].

Solution conductivity determines the charge density of the polymer solution, which in turn controls the repulsion and bending extent during electrospinning. Consequently, it affects the final mean fiber diameter. Uyar and Besenbacher [28] applied different grades of DMFs with slightly different solution conductivities as the solvent for Polystyrene polymer solutions, and they investigated its effect on the morphology of the nanofibers (presence of beads, nanofiber mean diameter). They found that the higher the conductivity, the smaller the diameter of the fibers.

Piezoelectric materials can usually be produced through three main steps (melting, mechanical stretching, and electric polarization) that have to be done in order to obtain the desired structure. Electrospinning has been shown to be a good alternative technique, thanks to the principles that form the basis of all electrohydrodynamic technologies: high electric potential neutralizes some stray ions in solutions, and charge imbalance occurs; then, when the repulsive forces exceed surface tension, an electrified liquid jet is ejected from the tip of the needle, solvent evaporates, and several electrical instabilities occur, causing the stretching of the jet and, finally, the solidification of nonwoven fibers. Consequently, the main advantage of the process is the ability to obtain mechanical stretching and polarization at the same time with a relatively high throughput.

Many works [29,30] have focused on improving the piezoelectricity of PVDF nanofibrous membranes by controlling the electrospinning parameters. Gee et al. [29] synthesized a set of membranes with systematically variable electrospinning parameters (the fraction between DMF and ACE, tip-to-collector distance (TCD), flow rate, and voltage setting), and they ranked parameters according to the contribution on the β-phase fraction: solvent > flow rate > TCD > voltage. Singh et al. [30] studied the effects of eight electrospinning parameters on β-phase content and gave a detailed explanation. However, the connection among parameters and the relative contribution to the β-phase fraction was not investigated. Accordingly, the present work is meant to study the effect of the solvent properties and electrospinning parameters on the morphology, the β-phase fraction, the crystallinity, and the piezoelectric voltage output of PVDF nanofibrous membranes. Eight solvents with suitable solubility and evaporation rates were selected to study the impacts of dipole moment on piezoelectric properties. Different voltages, feed rates, and distances were altered to present the effect of electrospinning parameters on piezoelectric properties. To the best of our knowledge, few papers have investigated the effect of solvents on the piezoelectric properties of PVDF electrospun nanofibrous membranes. In brief, this paper provides an effective and efficient strategy for regulating the piezoelectric properties of PVDF membranes by electrospinning: controlling solution solvent and process parameters.

## 2. Materials and Methods

### 2.1. Materials

PVDF (KYNAR 500) was purchased from Arkema (Colombes, France). Dimethyl sulfoxide (DMSO), N, N-dimethylformamide (DMF), acetone (ACE), and tetrahydrofuran (THF) were purchased from Sigma Aldrich (Burlington, MA, USA). All reagents were used as received without any further treatment.

### 2.2. Preparation of PVDF Solutions

To explore the effect of solvents on the PVDF nanofibrous membrane, a series of two solvents mixtures were prepared, as listed in Table 2. A total of 1 g PVDF powder was added into 6 g mixed solvent in a glass vial, and the solution was stirred for 8 h at room temperature (20 ± 3 °C).

The Hansen solubility parameters and physical properties (dipole moment and boiling point) of the mixed solvents are listed in Table 2. All results of the mixture are expressed in terms of the weight ratio of the individual pure components.

### 2.3. Preparation of PVDF Nanofibrous Membrane

**Figure 1:**
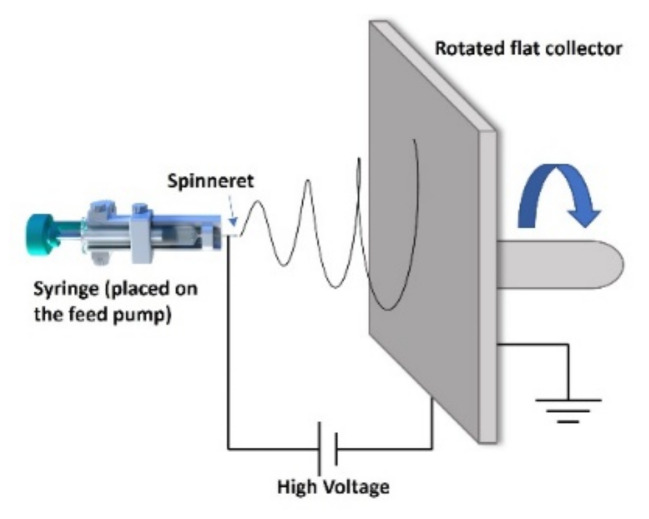
Schematic of the electrospinning setup utilized for the preparation of the PVDF nanofibrous membrane.

A 5 mL syringe loaded with PVDF solution was placed on a syringe pump with a feed rate of 0.5 mL h^−1^. A 27 G stainless needle with an inner diameter of 0.4 mm was used as a spinneret, and it was connected to a high-positive-voltage supply. A stainless rotated flat plate covered with aluminum foil was used as the collector. The applied voltage and the tip-to-collector distance were 10 kV and 15 cm, respectively. A schematic diagram of the electrospinning setup can be found in Figure 1.

The solution of PVDF dissolved in DMSO/ACE (2/1) solvent was electrospun with different electrospinning parameters (voltages, feed rates, and distances) equal to all the other operating variables in order to investigate the influence of the electrospinning parameters on the PVDF nanofibrous membrane.

The process parameters of each membrane are listed in Table 3. All electrospinning processes were carried out in an atmospheric environment (temperature: 20 ± 3 °C, humidity: 45 ± 5%). Then, the collected membranes were dried in an oven at 60 °C for 6 h to remove the remaining solvent.

### 2.4. Characterizations

The morphology of the membrane was observed by a scanning electron microscope (SEM) (JSM-6490, JEOL, Ltd., Tokyo, Japan) with a voltage of 15 kV, and all samples were sputtered with a thin Au layer before imaging. The mean diameter and standard deviation of nanofibers for each electrospun mat were obtained by randomly measuring 200 nanofibers from SEM images. The crystallinity of the PVDF membrane was analyzed by differential scanning calorimetry (DSC) (Q200, TA instruments, New Castle, DE, USA) using the heat-cool-heat procedure from 40 to 250 °C with a rate of 10 °C/min in a nitrogen atmosphere. The β-phase fraction of PVDF was analyzed by Fourier transform infrared spectroscopy (FTIR) (Nicolet Is50 spectrometer, Thermo Fisher Scientific, Waltham, MA, USA) in transmission mode, in the 1600–650 cm^−1^ wavenumber range (64 scans, 4 cm^−1^ resolution).

### 2.5. The Piezoelectric Analysis

**Figure 2:**
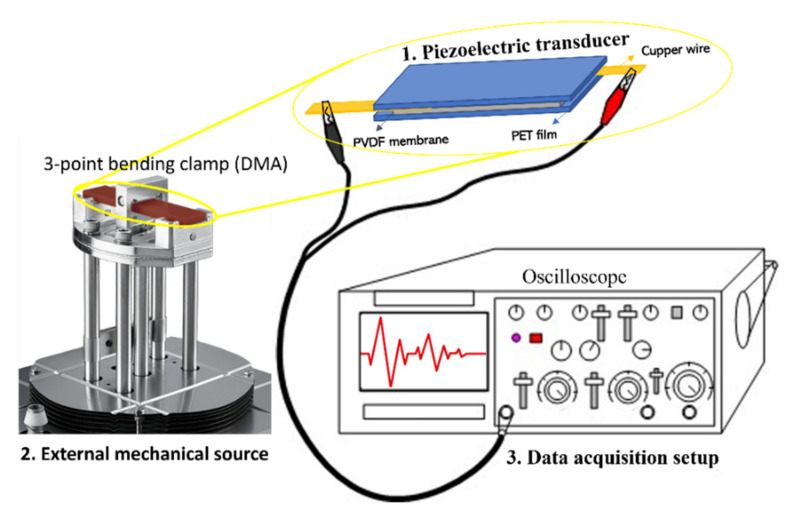
Schematic of the experimental setups utilized for piezoelectric analysis.

A piece of PVDF nanofibrous membrane (length × width × thickness: 60 × 16 × 0.6 mm^3^) was sandwiched between the conductive side of two PET films. Two copper wires were attached to the two sides of the PVDF membrane through the silver paint. Later, the PI tape was used to pack and protect the whole transducer. The transducer was fixed on a 3-point bending clamp for dynamic mechanical analysis (DMA) (Q800, TA instruments, New Castle, DE, USA), which can supply a regular and controllable strain. Another end of two copper wires from the transducer was connected to an oscilloscope (LT322, LeCroy, Chestnut Ridge, NY, USA), which worked as the acquisition setup. The assembly method of the transducer and the working mode for analysis are shown in Figure 2.

The same frequency (0.5 Hz) with different strains (1500, 2500, and 3000 um) and the same strain (2000 um) with different frequencies (0.25, 0.5, and 1 Hz) were applied on the transducer by DMA. The open-circuit voltage of the transducer under strain was recorded by the oscilloscope. Analyses under different conditions were performed twice at room temperature.

## 3. Results and Discussion

### 3.1. Effect of Solvent

**Figure 3:**
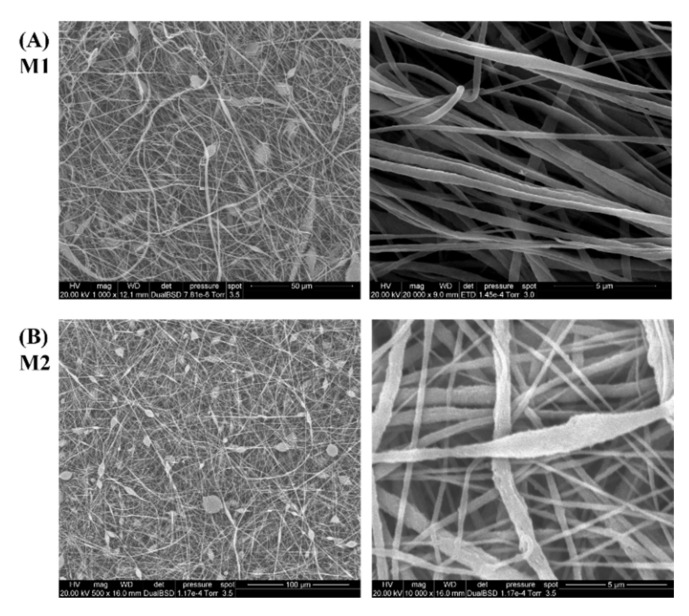
SEM images of PVDF nanofibrous membrane at two magnifications: (**A**) M1 (using DMF/ACE (2/1)) and (**B**) M2 (using DMF/THF (1/1)).

DMF/ACE (2/1) and DMF/THF (1/1) are the common solvents used to prepare PVDF solutions for electrospinning in the literature. Figure 3 shows the SEM images of PVDF membranes (M1 and M2) prepared using these two solvents. Beads, nanofibers with nonuniform morphology and various diameters, can be found in the membranes. It can be seen from the literature [31,32] that when these solvents are applied, similar phenomena frequently occur in the PVDF membrane.

**Figure 4:**
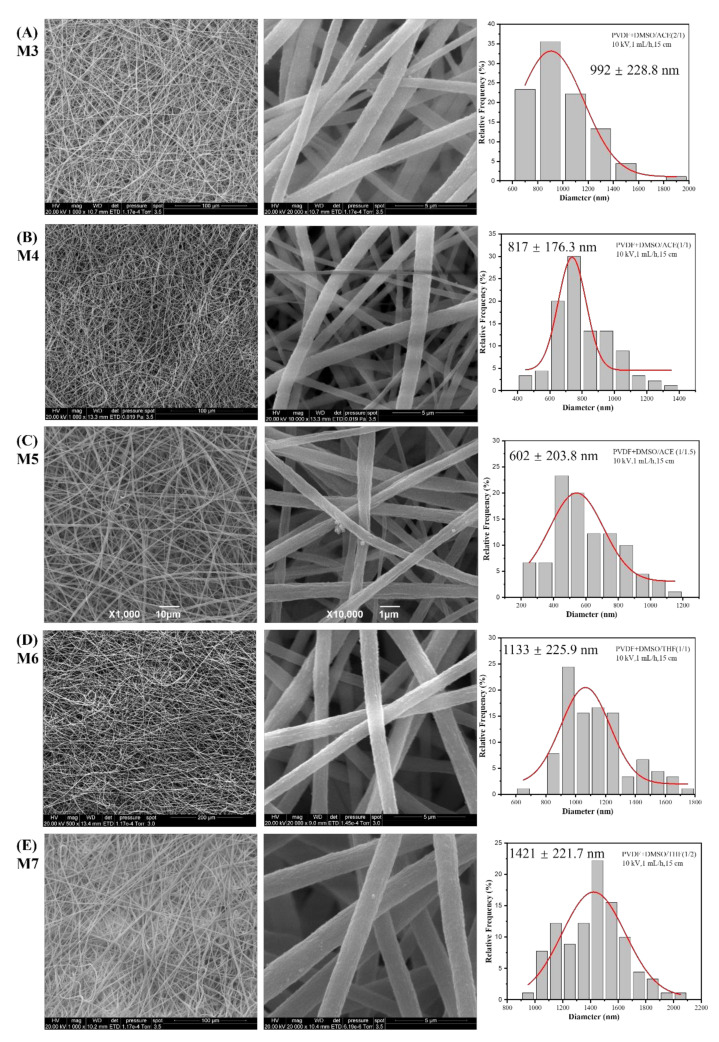
SEM images at two magnifications and the diameter histogram of PVDF nanofibrous membrane (**A**) M3 (using DMSO/ACE (2/1)), (**B**) M4 (using DMSO/ACE (1/1)), (**C**) M5 (using DMSO/ACE (2/3)), (**D**) M6 (using DMSO/THF (1/1)), and (**E**) M7 (using DMSO/THF (1/2)).

Figure 4 presents the SEM images at two magnifications and the diameter histogram of the PVDF nanofibrous membrane (M3-M7). From the SEM images at low magnification, it can be observed that these membranes consisted of nanofibers without defects, with morphology completely different from that of M1 and M2 in Figure 3. Another advantage of adopting DMSO as a solvent is its low toxicity, as emphasized by Russo et al. [33].

From the SEM images at high magnification, the smooth surface and uniform shape of the nanofibers were observed. The difference in average diameter among membranes can be attributed to the evaporation-stretching function during the electrospinning process. More specifically, the solvent with low boiling temperature was completely evaporated during electrospinning. Then, nanofibers with small diameter were gathered on the collector; otherwise, the nanofiber with residual solvent reached the collector and presented a large diameter. The stretching of nanofibers during the process was determined by not only the electrospinning parameters but also the solution conductivity; high stretching definitely leads to high elongation of the jet, as well as the formation of uniform fibers with a small diameter. ACE has higher solution conductivity than DMSO, followed by THF, so the conductivity of the solvents was increased from M3 to M4 and from M5 to M6. From the diameter of each membrane, it can be concluded that the conductivity of solution has a more pronounced effect on the fiber diameter than evaporation.

**Figure 5:**
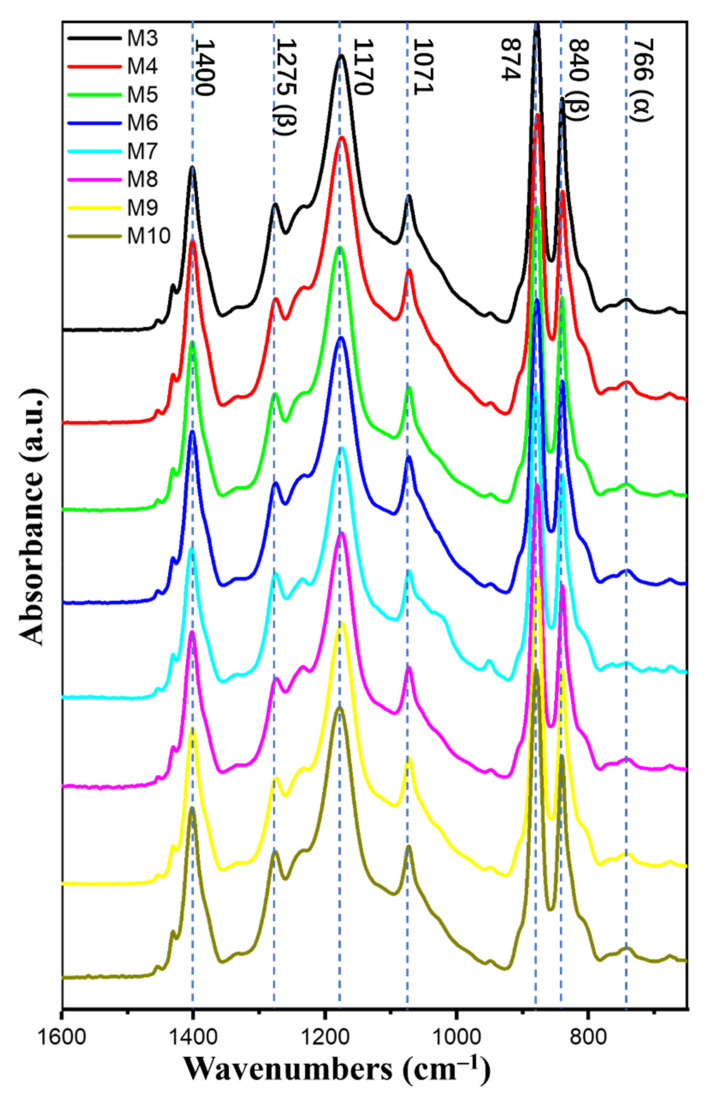
FTIR spectra of all PVDF nanofibrous membranes (M3-M10).

The FTIR spectra of all the samples are reported in Figure 5. The peaks at 1400, 1171, 1071, and 874 cm^−1^ were related to the CH_2_ wagging vibration, symmetrical stretching of -CF_2_, C-C asymmetric stretching, and CF_2_ symmetric stretching, respectively, and these are common bands for all the various PVDF phases. The peaks at 1275 and 840 cm^−1^ were attributed to CF out-of-plane deformation and CH_2_ rocking, and they were characteristic bands for β phase of PVDF. The peak at 766 cm^−1^ was raised from CF_2_ bending and skeletal bending, corresponding to the α phase [6,34,35,36].

**Figure 6:**
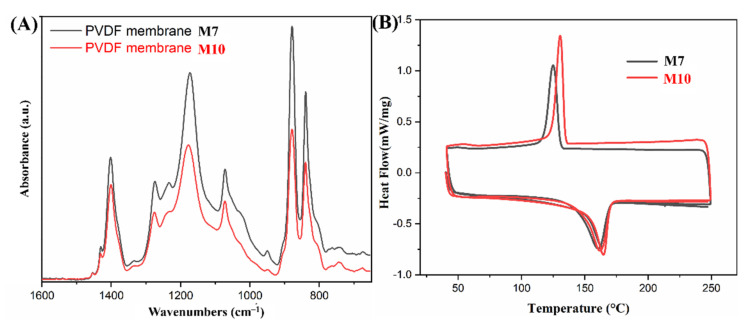
(**A**) FTIR spectra and (**B**) DSC curves of PVDF nanofibrous membranes (M7 and M10).

To clearly observe the difference of curves and simply explain the calculation of the β-phase fraction ($F\left(\beta \right)$), only the FTIR spectra of M7 and M10 are presented in Figure 6A. $F\left(\beta \right)$ can be calculated using the Beer–Lambert Equation (2).

(2) $$F\left(\beta \right)=\frac{A_{\beta }}{A_{\beta }+\frac{K_{\beta }}{K_{\alpha }}A_{\alpha }}\;\times \;100\%.$$

where K_α_ (6.1 × 10^4^ cm^2^ mol^−1^) and K_β_ (7.7 × 10^4^ cm^2^ mol^−1^) are the absorption coefficients at 766 and 840 cm^−1^, respectively; and A_α_ and A_β_ are the absorbencies at 766 and 840 cm^−1^, respectively [34].

The average β-phase fractions at five different places on each membrane are summarized in Table 3. All electrospun membranes had a relatively high β-phase content (above 80%) compared with the membrane prepared by casting because of the voltage field and the stretch during the electrospinning process. On the other hand, a high β-phase fraction was obtained in the PVDF electrospun membrane when a solvent with a high dipole moment was used.

Figure 6B is the DSC curves of M7 and M10. Crystallinity is calculated according to Equation (3):

(3) $$X_{c}=\frac{\Delta H}{\Delta H_{m}\cdot \varphi }\times 100\%$$

where ΔH is the fusion enthalpy of the PVDF membrane obtained from the DSC curve, $\Delta H_{m}$ (104.7 J g^−1^) is the fusion enthalpy of PVDF with 100% crystallinity, and φ is the PVDF weight fraction. The endothermic peak of the second heating process was used to calculate the crystallinity.

The crystallinity summarized in Table 3 is the average of the three analyses. More than 50% crystallinity can be reached in the PVDF membrane through electrospinning. Furthermore, the relation between crystallinity and solvent showed a trend similar to that between β-phase content and solvent: the higher the dipole moment of the solvent, the higher the crystallinity of the PVDF membrane.

Consequently, the solvent affects not only the β-phase fraction but also the crystallinity. Adopting a solvent with a high dipole moment can produce a PVDF membrane with a high β-phase content and high crystallinity. This effect on cast films has been previously reported with the explanation that solvents with a high dipole moment can enhance the end-to-end length and lead to the regular orientation of PVDF chains, which in turn results in dipole alignment and good piezoelectric properties of PVDF membranes. [27,37,38]

### 3.2. Effect of Electrospinning Parameters

The effects of electrospinning parameters on the crystallinity and β-phase content of PVDF membranes have been reported in published works [29,30]. Here, PVDF electrospun membranes (M3, M8–M10) with different voltages, feed rates, and distances were prepared, and we confirmed that the electrospinning parameters certainly influence the piezoelectric properties of PVDF membranes.

**Figure 7:**
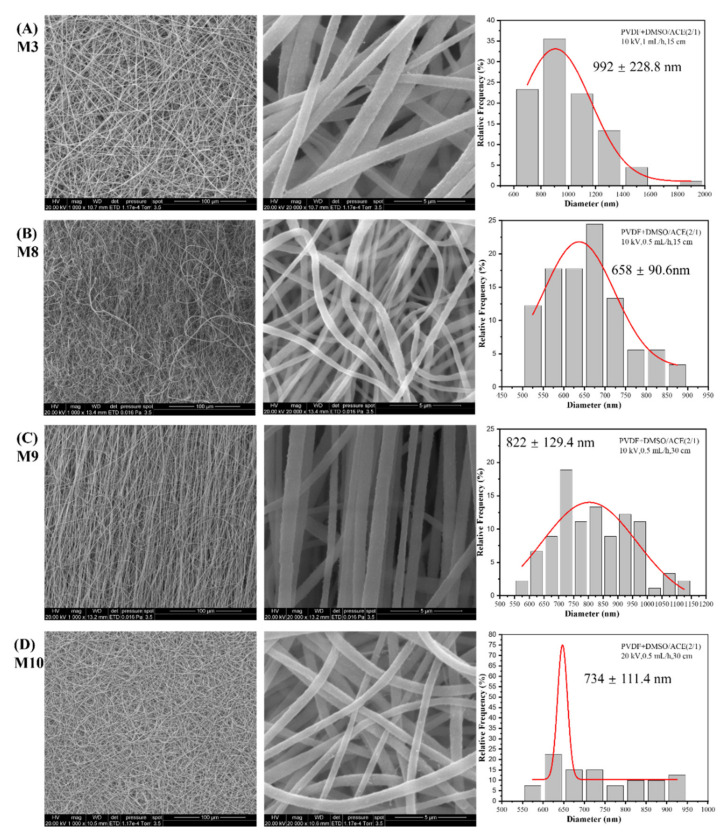
SEM images at two magnifications and the diameter histogram of PVDF nanofibrous membrane (**A**) M3 (using 10 kV, 1 mL/h, 15 cm), (**B**) M8 (using 10 kV, 0.5 mL/h, 15 cm), (**C**) M9 (using 10 kV, 0.5 mL/h, 30 cm), and (**D**) M10 (using 20 kV, 0.5 mL/h, 30 cm).

Figure 7 shows SEM images at two magnifications and the nanofiber diameter histogram of PVDF membranes prepared with different electrospinning parameters. Obviously, when the same solvent but different electrospinning parameters were adopted, uniform and bead-free PVDF membranes were synthesized. The ratio of voltage-to-distance can be regarded as the voltage field intensity. From M3 to M8, the diameter of nanofibers reduced due to the decrease in feed rate, which meant the same voltage field intensity was applied on less solution, or the same amount of solution was applied under higher voltage field intensity. From M8 to M9, a longer distance led to a lower voltage field intensity, which resulted in an increase in nanofiber diameter. The diameter decreased from M9 to M10 because the higher field intensity, generated by the higher voltage, provides a stronger force.

The β-phase fraction and the crystallinity are summarized in Table 3. The influence of electrospinning parameters on the β-phase fraction and crystallinity was present but insignificant, and it was even more difficult to detect any trends. Therefore, no more explanation about the influence can be given here.

As consequence, the solvent was the main factor that manipulated the morphology of the PVDF membranes, whereas electrospinning parameters had influences on the morphology (e.g., diameter, bead) of nanofibers. The effects of electrospinning parameters can be explained as follows: the voltage applies a stretching force on the jet from the needle, the feed rate determines the shape of the Taylor cone on the tip of the needle, and the distance affects the stretching time before reaching the collector. There are internal relations between these parameters, so it is impossible to discuss the impact of an individual parameter or attribute results to a single parameter.

### 3.3. Piezoelectric Analysis

**Figure 8:**
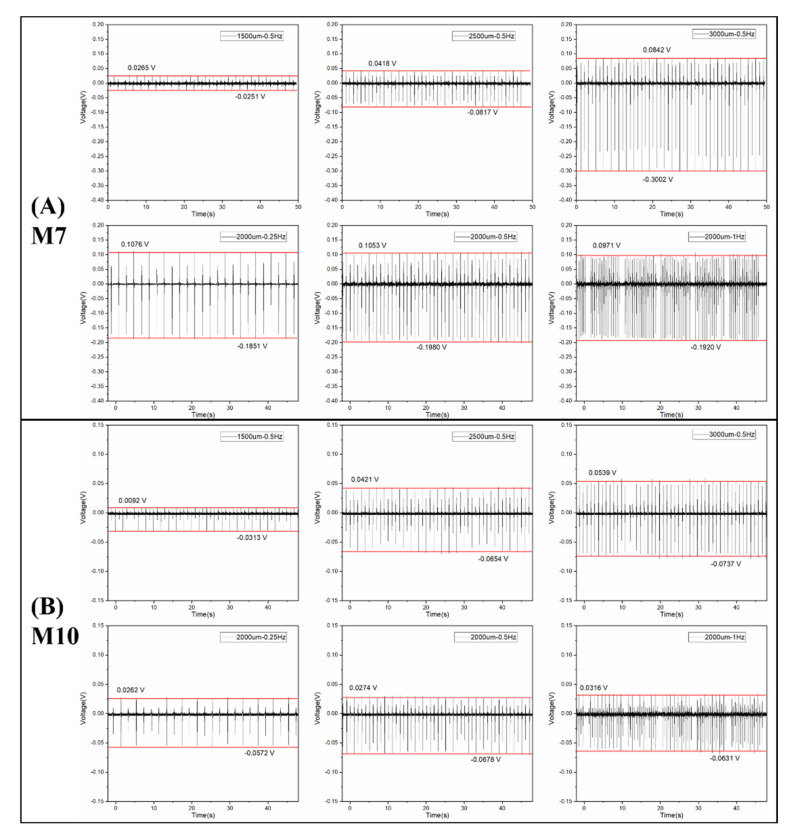
The open-circuit voltage of the transducers is made of PVDF membranes (**A**) M7 and (**B**) M10 as a function of time under different amplitudes and frequencies.

M7 and M10 were selected for piezoelectric measurements because they presented the lowest and the highest crystallinity and β phase, respectively, among all the membranes. The open-circuit voltage as a function of time under various external strains is shown in Figure 8, and the piezoelectric voltage outputs (the value between the highest and lowest voltage) are summarized in Table 4. The voltage output increased with increased amplitude and remained stable with the change in frequency, as previously observed by Chen et al. [39]. With the progress of the piezoelectric measurements (especially after the measurements at 3000 um–0.5 Hz), the transducers became more flexible and therefore more easily stretched under the same strain with respect to the beginning conditions. Consequently, the voltage output of the transducer at 2000 um–0.5 Hz was higher than that at 2500 um–0.5 Hz.

Comparing the voltage output of the transducers made of M7 and M10, it can be seen that the voltage outputs of M10 were always higher than those of M7 under the different external strains. Hence, M10 had a higher piezoelectric response than M7, which corresponded to the higher crystallinity and higher β-phase fraction of M10.

## 4. Conclusions

This work provides a new insight for the preparation of PVDF electrospun membranes with piezoelectric properties based on dipole moment of solvents. The morphology, nanofiber diameter, crystallinity, and β-phase fraction of the synthesized PVDF membrane were studied, and piezoelectric analysis of the transducers made of two PVDF membranes was carried out. When DMSO with good solubility for PVDF and low toxicity was used, membranes with good morphology were obtained. When DMSO/ACE (2/1) with high dipole moment was used as the solvent, PVDF electrospun membranes exhibited a higher crystallinity, β-phase fraction, and piezoelectric output than that prepared with DMSO/THF (1/2). Based on the presented analysis, we found that solvents with a high dipole moment can improve piezoelectric properties, and the evaporation rate and solvent conductivity can influence nanofiber diameter. On the other hand, electrospinning parameters can also control nanofiber diameter and piezoelectric properties during the electrospinning process, although the effect of the solvents is much more straightforward. Therefore, selecting a proper solvent can be considered a simple method to control the piezoelectric performance of PVDF membranes.

## Figures and Tables

**Table 1:** The Hansen solubility parameters and physical properties of PVDF and various solvents.

Solvent	*δ_d_*	*δ_p_*	*δ_h_*	*R_a_*	RED (*R_a_/R_o_*) ^1^	Dipole Moment	Boiling Point
Unit	MPa^1/2^	MPa^1/2^	MPa^1/2^	MPa^1/2^		D	°C
PVDF	17.2	12.5	9.2	0			
DMSO	18.4	16.4	10.2	4.68	0.936	3.96	189
DMF	17.4	13.7	11.3	2.45	0.49	3.82	153
ACE	15.5	10.4	7	4.56	0.912	2.85	56
THF	16.8	5.7	8	6.95	1.39	1.63	65

^1^ R_o_ of PVDF adopted = 5, as suggested in the previous work [22].

**Table 2:** Hansen solubility parameters and physical properties of the mixed solvents.

Solvent	δ_d_	δ_p_	δ_h_	R_a_	RED (R_a_/R_o_)	Dipole Moment	Boiling Point
unit	MPa^1/2^	MPa^1/2^	MPa^1/2^	MPa^1/2^		D	°C
DMF/ACE (2/1)	16.7	12.6	9.8	1.10	0.70	3.50	120.7
DMF/THF (1/1)	17.1	9.7	9.7	2.84	0.55	2.73	109.0
DMSO/ACE (2/1)	17.4	14.4	9.1	1.96	0.39	3.59	144.7
DMSO/ACE (1/1)	16.9	13.4	8.6	1.19	0.24	3.41	122.6
DMSO/ACE (2/3)	16.7	12.8	8.3	4.61	0.92	3.29	109.3
DMSO/THF (1/1)	17.6	11.1	9.1	1.66	0.33	2.80	127.0
DMSO/THF (1/2)	17.3	9.3	8.7	3.30	0.66	2.41	106.3

**Table 3:** Process parameters (solvent and electrospinning properties), nanofiber diameter, crystallinity, and β-phase fraction of the PVDF membranes.

Num.	Solvents	Dipole Moment	Boiling Point	Electrospinning Parameters	Diameter	Crystallinity (DSC)	β Phase (FTIR)
	unit	D	°C		nm	%	%
M1	DMF/ACE (2/1)	3.50	120.7	10 kV, 1 mL/h, 15 cm	- ^1^	-	-
M2	DMF/THF (1/1)	2.73	109.0		-	-	-
M3	DMSO/ACE (2/1)	3.59	144.7		992 ± 228.8	52.30	87.49
M4	DMSO/ACE (1/1)	3.41	122.6		817 ± 176.3	51.61	86.88
M5	DMSO/ACE (2/3)	3.29	109.3		602 ± 203.8	51.25	86.36
M6	DMSO/THF (1/1)	2.80	127.0		1133 ± 225.9	50.27	84.70
M7	DMSO/THF (1/2)	2.41	106.3		1421 ± 221.7	48.67	81.91
M8	DMSO/ACE (2/1)	3.59	144.7	10 kV, 0.5 mL/h, 15 cm	658 ± 90.6	50.46	85.36
M9				10 kV, 0.5 mL/h, 30 cm	822 ± 129.4	50.37	86.42
M10				20 kV, 0.5 mL/h, 30 cm	734 ± 111.4	52.36	88.01

^1^ The properties of M1 and M2 were not studied further due to poor morphological quality.

**Table 4:** Piezoelectric voltage outputs of transducers made of M7 and M10 under different amplitudes and frequencies.

Sample		M7	M10
Different amplitude	1500 um–0.5 Hz	39.1 mV	48.8 mV
	2500 um–0.5 Hz	106.4 mV	111.8 mV
	3000 um–0.5 Hz	119.9 mV	369.7 mV
Different frequency	2000 um–0.25 Hz	83.4 mV	285.7 mV
	2000 um–0.5 Hz	88.2 mV	298.6 mV
	2000 um–1 Hz	94.7 mV	289.1 mV

## Data Availability

Not applicable.
